# The Johns Hopkins Hospital Reports

**Published:** 1900-03

**Authors:** 


**The Johns Hopkins Hospital Reports.** Vol. VIII. Nos. 1–2. *On the rôle of insects, arachnids and myriapods, as carriers in the spread of bacterial and parasitic diseases of man and animals.* By GEORGE H. F. NUTTALL, M.D., Ph.D. Pp. 152. Baltimore: The Johns Hopkins Press. 1899.

This is a most fascinating collection of the facts and literature relating to a little known province of natural history. Few readers who take up the story will be content till they have perused it from end to end. It contains much which has been forgotten and much which is only accessible elsewhere in second-hand reports. The writer has brought together from scattered sources an immense number of records, facts and theories as to the disease-carrying agency of flies, fleas, ants, ticks, bugs, and mosquitoes.

He shows that in some cases the experimental work done in support of these statements is exceedingly scanty, and many of them must be doubted; but in others, such as the life history of the mosquito, our knowledge has been carried beyond the region of doubt, and placed on a firm foundation. With regard to the mosquito, so much has been done in the last twelve months that his well-told tale is already a little out of date, but he has done well to bring out the remarkable anticipations of Dr. King, who preceded the present workers. The late American war, too, led to a copious literature on the spread of typhoid by flies. He finds that the conveyance of anthrax by flies is possible, but probably rare; the experimental evidence, too, is against the action of fleas and bugs. Both the latter have been accused of spreading the plague in India, but though there are some grounds for the belief, more evidence is needed. However, the importance of flies in carrying cholera germs, and scattering them amongst articles of food is not to be doubted, and indeed it is clear that typhoid is easily disseminated in the same way, when care is not taken to disinfect the excreta. The sections on the marvellous conveyance of the filaria, the Texas cattle fever, and on the part played by the Tsetse fly, are fascinating in the extreme, and the copious facts as to the life history of mosquitos, collected from out of the way records, should be of great value in the present investigations.

